# Association between triglyceride-glucose related indices and mortality among individuals with non-alcoholic fatty liver disease or metabolic dysfunction-associated steatotic liver disease

**DOI:** 10.1186/s12933-024-02343-7

**Published:** 2024-07-04

**Authors:** Qingling Chen, Pingping Hu, Xiaoxue Hou, Ye Sun, Mengfan Jiao, linya Peng, Zixing Dai, Xizhi Yin, Rui Liu, Yuwen Li, Chuanlong Zhu

**Affiliations:** 1 Department of Infectious Disease, The First Affiliated Hospital of Nanjing Medical University, Nanjing, 210029 China; 2 NHC (National Health Commission of the People’s Republic of China) Key Laboratory of Tropical Disease Control, The Second Affiliated Hospital of Hainan Medical University, Haikou, 570216 China; 3 Department of Pediatrics, The First Affiliated Hospital of Nanjing Medical University, Nanjing, 210029 China

**Keywords:** Non-alcoholic fatty liver disease, Metabolic dysfunction-associated steatotic liver disease, TyG index, TyG-BMI index, TyG-WC index, Mortality, NHANES III

## Abstract

**Background:**

The prognostic value of triglyceride-glucose (TyG) related indices in non-alcoholic fatty liver disease (NAFLD) or metabolic dysfunction-associated steatotic liver disease (MASLD) is still unclear. This study aimed to determine the associations between TyG-related indices and long-term mortality in this population.

**Methods:**

The data came from the National Health and Nutrition Examination Survey (NHANES III) and National Death Index (NDI). Baseline TyG, TyG combining with body mass index (TyG-BMI), and TyG combining with waist circumference (TyG-WC) indices were calculated, and mortality status was determined through 31 December 2019. Multivariate Cox and restricted cubic spline (RCS) regression models were performed to evaluate the relationship between TyG-related indices and long-term mortality among participants with NAFLD/MASLD. In addition, we examined the association between TyG-related indices and all-cause mortality within subgroups defined by age, sex, race/ethnicity, and fibrosis-4 index (FIB-4).

**Results:**

There were 10,390 participants with completed ultrasonography and laboratory data included in this study. NAFLD was diagnosed in 3672/10,390 (35.3%) participants, while MASLD in 3556/10,390 (34.2%) amongst the overall population. The multivariate Cox regression analyses showed high levels of TyG-related indices, particularly in TyG-BMI and TyG-WC indices were significantly associated with the all-cause mortality, cardiovascular mortality, and diabetes mortality in either NAFLD or MASLD. The RCS curves showed a nonlinear trend between three TyG-related indices with all-cause mortality in either NAFLD or MASLD. Subgroup analyses showed that TyG-BMI and TyG-WC indices were more suitable for predicting all-cause mortality in patients without advanced fibrosis.

**Conclusion:**

Our study highlights the clinical value of TyG-related indices in predicting the survival of the NAFLD/MASLD population. TyG-BMI and TyG-WC indices would be the surrogate biomarkers for the follow-up of the population without advanced fibrosis.

## Introduction

Non-alcoholic fatty liver disease (NAFLD) is recognized as the most prevalent chronic liver disease, impacting about 30% of the global individuals, with the potential to progress to advanced hepatic fibrosis and end-stage liver diseases eventually [1, 2]. In June 2023, a multisociety committee of international experts was gathered and adopted new terminology to reduce stigma and increase awareness of the disease [3]. A new terminology, steatotic liver disease (SLD), replaces the term “fatty liver disease” as the umbrella term. Under subgroups of SLD, NAFLD has been renamed as metabolic dysfunction-associated steatotic liver disease (MASLD) [3].

NAFLD/MASLD is closely related to metabolic syndrome (MetS) and its associated components of hypertension, type 2 diabetes mellitus, dyslipidaemia and obesity [4, 5]. Insulin resistance (IR) refers to a reduced efficiency of insulin in promoting glucose utilization, which is a prominent feature of MetS [6]. As the gold standard for measuring IR, the hyperinsulinemic euglycemic clamp test is complex and invasive, making it unsuitable for clinical research [7]. The homeostatic model assessment of IR (HOMA-IR), calculated from fasting blood glucose (FBG) and insulin concentrations, is a validated alternative evaluation index [8]. However, since circulating insulin concentrations are rarely measured in primary care, another alternative evaluation index for IR has been developed, such as the triglyceride-glucose (TyG) index [9]. Some studies have found that TyG index is associated with new-onset NAFLD and the degree of hepatic steatosis [10–12]. Other studies have found that the TyG index is associated with all-cause and cardiovascular mortality in different populations, including coronary heart disease and hypertension, ischemic stroke, and general population [13–15]. Further more, TyG combined with adiposity indices, such as TyG combining with body mass index (TyG-BMI), and TyG combining with waist circumference (TyG-WC) indices may be better than the single TyG index [16, 17]. However, few studies have evaluated the correlation between the TyG-related indices and mortality in patients with NAFLD/MASLD.

This study linked the National Health and Nutrition Examination Survey (NHANES) and National Death Index (NDI) data to evaluate the associations between TyG-related indices and long-term all-cause/cause-specific mortalities among participants with NAFLD/MASLD. Here, we aim to identify whether TyG-related indices are valuable predictors of the survival status in this population.

## Methods

### Study population

This study is based on an analysis of the NHANES III (1988–1994, the National Center for Health Statistics, Centers of Disease Control and Prevention), which uses a national, multistage, stratified cluster design to make a representative sample of the noninstitutionalized civilian population in the United States. Of 20,050 participants (20–74 years) from NHANES III, we excluded those with missing data on mortality (*n* = 451), pregnant women (*n* = 264), participants who were not eligible for an ultrasound examination (age exclusion) or whose ultrasound was ungradable or missing (*n* = 5705), participants missing data on TyG-related indices (*n* = 3240), leading to the final study’s sample of 10,390 participants. The NHANES survey was approved by the institutional review board of the National Center for Health Statistics, and all participants provided written informed consent.

### Definitions of SLD, NAFLD and MASLD

The presence of hepatic steatosis was determined by the ultrasonic examinations with the Toshiba Sonolayer SSA-90 A (Toshiba America Medical Systems, Tustin, CA) [18]. Those with hepatic steatosis (including mild, moderate, and severe cases) were diagnosed as SLD. NAFLD was defined as having SLD in the absence of excessive alcohol intake (≥ 30 g/d for males and ≥ 20 g/d for females) or positive hepatitis B and C viral infection [19]. MASLD was defined as having SLD plus the presence of 1 of the following cardiometabolic adult criteria without other causes of hepatic steatosis or excessive alcohol consumption (≥ 30 g/d for males and ≥ 20 g/d for females) [3]. Cardiometabolic adult criteria were defined as the following: (1) BMI ≥ 25 kg/m^2^ (23 kg/m^2^ for Asia) or WC > 94 cm for males and > 80 cm for females; (2) FBG ≥ 5.6 mmol/L (100 mg/dL) or 2-hour post-load glucose levels ≥ 7.8 mmol/L (140 mg/dL) or glycated hemoglobin (HbA1c) ≥ 5.7% (39 mmol/L) or type 2 diabetes or treatment for type 2 diabetes; (3) blood pressure ≥ 130/85 mmHg or specific antihypertensive drug treatment; (4) plasma triglycerides (TG) ≥ 1.70 mmol/L (150 mg/dL) or lipid lowering treatment; and (5) plasma high-density lipoprotein cholesterol (HDL-C) ≤ 1.0 mmol/L (40 mg/dL) for males and ≤ 1.3 mmol/L (50 mg/dL) for females or lipid lowering treatment [3].

### Definitions of TyG-related indices

The TyG index is a surrogate of IR based on FBG and TG levels. At baseline, participants’ FBG and TG were measured. The TyG, TyG-BMI, and TyG-WC indices were calculated according to the following formulas: TyG index = Ln [fasting TG (mg/dL) × FBG (mg/dL)/2]; TyG-BMI index = TyG index × BMI; TyG-WC index = TyG index × WC [20, 21].

### Assessment of covariates

Covariates for this study included age, sex, race/ethnicity, income level, marital status, educational level, smoking, physical activity, BMI, WC, diabetes, hypertension, TG, HDL-C, HbA1c, FBG, fasting insulin, HOMA-IR, NAFLD Fibrosis Score (NFS), and fibrosis-4 index (FIB-4).

Race/ethnicity of participants were divided into four categories: non-Hispanic white, non-Hispanic black, Mexican-American, and other race. A low income level was defined as poverty-income ratio (PIR) < 1.3 [22]. Sedentary lifestyle was defined if participants answered ‘no’ to all questions regarding any physical activities listed over the last month: jogging or running, swimming, bicycling, aerobics, callisthenics exercises, other dancing, garden or yard work, weight lifting or other sports activities [23]. BMI was calculated by dividing body weight (kg) by the square of height (m^2^) [24]. It was defined as diabetes if the FBG level was ≥ 126 mg/dL, HbA1c level was ≥ 6.5% and/or self-reported doctor diagnosis and/or anti-diabetic treatment [25]. It was defined as hypertension if the systolic blood pressure was ≥ 140 mmHg or the diastolic blood pressure was ≥ 90 mmHg and/or self-reported doctor diagnosis and/or antihypertensive treatment [23]. The HOMA-IR is another surrogate of IR and was calculated as follows: HOMA-IR = FBG (mmol/L) × fasting insulin (µU/mL)/22.5 [26]. Non-invasive liver fibrosis assessment included NFS and FIB-4, which were calculated as follows: NFS = − 1.675 + 0.037 × age (years) + 0.094 × BMI (kg/m^2^) + 1.13 × impaired fasting glucose/diabetes (yes = 1, no = 0) + 0.99 × AST/ALT ratio − 0.013 × platelet (× 10^9^/L) − 0.66 × albumin (g/dL), FIB-4 index = [age (years) × AST (U/L)] / [platelet (×10^9^/L) × ALT (U/L) ^ (1/2)] [27, 28]. For the diagnosis of advanced fibrosis, NFS and FIB-4 cut-off values were − 1.455 and 1.3 respectively [29].

### Ascertainment of mortality

The NHANES III data are linked to death records from NDI to ascertain all-cause and cause-specific mortality. The length of survival for each participant was determined by the time period between the date of NHANES III baseline examination and the date of death or 31 December 2019, whichever came first. Mortality from any cause was defined as all-cause mortality. International Classification of Diseases, Tenth Revision (ICD-10) was used to define cause-specific mortality. Specifically, cardiovascular mortality refers to deaths caused by major cardiovascular disease (CVD) and cerebrovascular diseases (codes: I00–I09, I11, I13, I20–I51 and I60–I69). Similarly, diabetes mortality refers to deaths caused by diabetes (codes: E10–E14).

### Statistical analysis

The baseline characteristics and cumulative mortality were reported as the median (interquartile range) for continuous variables, while unweighted frequency counts and weighted percentages were used for categorical variables. In order to compare the characteristics of the groups, we employed the Mann-Whitney test for continuous variables and the Rao-Scott chi-square test for categorical variables.

Cox proportional hazards models were utilized to estimate hazard ratios (HR) and 95% confidence intervals (CI) for the association of TyG-related indices with all-cause and cause-specific mortality among participants with NAFLD/MASLD. We used two models with progressive degrees of adjustment: model 1 adjusted for age, sex, and race/ethnicity; model 2 further adjusted for income, marital status, education, smoking, sedentary lifestyle, diabetes, hypertension, plasma HDL-C, and FIB-4.

To further explore the dose-effect correlations between TyG-related indices with all-cause mortality in NAFLD/MASLD, restricted cubic spline (RCS) regression models were employed. In addition, we examined the association between TyG-related indices and all-cause mortality within subgroups defined by age, sex, race/ethnicity, and FIB-4 among this population.

All statistical analyses were performed using the R software (version 4.3.3; R Foundation for Statistical Computing, Vienna, Austria), incorporating survey weights from NHANES III’s complex survey design through the utilization of the survey package. To determine a more rigorous interpretation of the associations between TyG, TyG-BMI, and TyG-WC with the survival of the NAFLD/MASLD population, the Bonferroni-corrected *p*-value was used in this study [30]. Thus, the significance level *p* = 0.05 was divided by three, which provided a significance level corrected for multiple testing: *p* = 0.017.

## Results

### Baseline characteristics of the participants

A total of 10,390 cases with completed ultrasonography and laboratory data were identified from the NHANES III database. NAFLD was diagnosed in 3672/10,390 (35.3%) participants, while MASLD in 3556/10,390 (34.2%) amongst the overall population. The comparison of clinical characteristics between NAFLD and Non-NAFLD, as well as MASLD and Non-MASLD group is illustrated in Table 1. Compared to individuals without NAFLD/MASLD, patients with the diagnosis of NAFLD/MASLD, irrespective of which criteria were used, were more likely to be older, Mexican American, married, higher metabolic indexes (including BMI, WC, TG, HbA1c, glucose, insulin, HOMA-IR, and TyG-related indices), lower HDL-C, higher levels of non-invasive liver fibrosis scores, and more likely to have low income, high sedentary lifestyle, diabetes, and hypertension, while they were less likely to have a higher education or be a current smoker (*p* < 0.017). There was no significant difference in the sex between adults with NAFLD and Non-NAFLD.

**Table 1:** Baseline characteristics of participants, stratified by NAFLD or MASLD status: NHANES III (1988–1994)

Characteristics	NAFLD (*n* = 3672 )	Non-NAFLD (*n* = 6718)	*p* value	MASLD (*n* = 3556)	Non-MASLD (*n* = 6834)	*p* value
Age (years)	45.0 (33.0, 61.0)	39.0 (39.0, 55.0)	<0.001	46.0 (35.0, 61.0)	38.0 (28.0, 54.0)	<0.001
Male, n (%)	1764 (51.6)	3146 (48.5)	0.246	1743 (53.6)	3167 (47.6)	0.01
Race/ethnicity, n (%)			<0.001			<0.001
Non-Hispanic white	1315 (67.2)	2563 (69.9)		1249 (65.9)	2629 (70.4)	
Non-Hispanic black	964 (12.4)	2234 (15.5)		923 (12.6)	2275 (15.3)	
Mexican-American	1236 (14.5)	1563 (8.3)		1225 (15.1)	1574 (8.2)	
Other race	157(5.8)	358 (6.3)		159 (6.3)	356 (6.0)	
Low income, n (%)	1302 (22.0)	2174 (19.1)	0.002	1290 (23.4)	2182 (18.5)	<0.001
Married, n (%)	2265 (68.2)	3738 (62.9)	<0.001	2213 (68.5)	3790 (62.9)	<0.001
College degree, n (%)	392 (19.4)	1020 (24.4)	<0.001	356 (17.7)	1056 (25.1)	<0.001
Current smoker, n (%)	873 (22.6)	2039 (30.1)	<0.001	857 (22.9)	2055 (29.8)	<0.001
Sedentary lifestyle, n (%)	1262 (26.4)	1847 (19.7)	<0.001	1265 (27.3)	1844 (19.5)	<0.001
BMI (kg/m^2^)	28.7 (24.9, 33.1)	25.4 (22.6, 28.7)	<0.001	29.2 (25.7, 33.4)	25.1 (22.4, 28.5)	<0.001
WC (cm)	99.0 (88.2, 109.0)	89.0 (80.0, 97.9)	<0.001	100.1 (90.7, 109.6)	88.3 (79.1, 97.4)	<0.001
Diabetes, n (%)	704 (15.6)	475 (5.3)	<0.001	722 (17.0)	457 (4.9)	<0.001
Hypertension, n (%)	1528 (38.9)	1922 (25.4)	<0.001	1580 (42.3)	1870 (24.1)	<0.001
TG (mg/dL)	137.5 (89.0, 209.0)	97.0 (69.0, 140.0)	<0.001	143.0 (95.0, 215.0)	95.0 (68.0, 137.0)	<0.001
HDL-C (mg/dL)	45.0 (37.0, 54.0)	50.0 (42.0, 61.0)	<0.001	44.0 (37.0, 53.0)	51.0 (42.0, 61.0)	<0.001
HbA1c (%)	5.5 (5.1, 5.9)	5.3 (5.0, 5.6)	<0.001	5.5 (5.1, 5.9)	5.3 (5.0, 5.6)	<0.001
Glucose (mg/dL)	95.8 (88.7, 105.8)	91.9 (86.4, 98.6)	<0.001	96.7 (89.7, 107.2)	91.6 (86.2, 98.2)	<0.001
Insulin (uU/mL)	12.23 (8.02, 19.44)	8.12 (5.85, 11.81)	<0.001	12.81 (8.53, 20.22)	7.95 (5.78, 11.52)	<0.001
HOMA-IR	3.00 (1.85, 5.15)	1.85 (1.30, 2.80)	<0.001	3.16 (1.98, 5.40)	1.81 (1.28, 2.72)	<0.001
TyG index	8.84 (8.34, 9.33)	8.40 (8.05, 8.83)	<0.001	8.88 (8.42, 9.37)	8.37 (8.03, 8.80)	<0.001
TyG-BMI index	260.11 (215.54,)	215.14 (186.17, 249.09)	<0.001	264.59 (224.74, 306.62)	212.92 (183.37, 247.23)	<0.001
TyG-WC index	888.01 (758.99,1002.91)	752.18 (656.87,850.9)	<0.001	900.35 (786.69, 1012.19)	744.92 (647.69, 844.88)	<0.001
NFS	−2.05 (−3.09, −0.86)	−2.47 (−3.34, −1.45)	<0.001	−1.94 ( −3.00, −0.77)	−2.50 ( −3.36, −1.50)	<0.001
NFS >-1.455, n (%)	1339 (33.8)	1690 (23.0)	<0.001	1368 (36.4)	1661 (22.0)	<0.001
FIB-4	0.82 (0.56, 1.24)	0.75 (0.52, 1.15)	<0.001	0.85 (0.58, 1.27)	0.74 (0.51, 1.13)	<0.001
FIB-4 > 1.3, n (%)	806 (22.3)	1250 (17.5)	<0.001	830 (24.0)	1226 (16.8)	<0.001
*Cumulative mortality, n (%)*						
All cause	1466 (37.6)	2062 (27.3)	<0.001	1508 (40.7)	2019 (26.0)	<0.001
Cardiovascular specific	391 (9.6)	546 (6.7)	<0.001	398 (10.4)	539 (6.4)	<0.001
Diabetes specific	70 (1.6)	62 (0.6)	<0.001	76 (1.8)	56 (0.5)	<0.001

Data are displayed as the median (interquartile range) or unweighted frequency counts (weighted percentage) as appropriate. The Mann-Whitney test for continuous variables and the Rao-Scott chi-square test for categorical variables were used in this analysis

BMI, body mass index; FIB-4, fibrosis-4 index; HbA1c, glycated hemoglobin; HDL-C, high-density lipoprotein cholesterol; HOMA-IR, homeostasis model assessment of insulin resistance; MASLD, metabolic dysfunction-associated steatotic liver disease; NAFLD, non-alcoholic fatty liver disease; NFS, NAFLD Fibrosis Score; TG, triglycerides; TyG, triglyceride-glucose; WC, waist circumference

### Mortality analysis for patients with NAFLD/MASLD

When NAFLD criteria was used, a total of 1466 (37.6%) deaths occurred during the median follow-up of 26.4 (interquartile range: 21.5–27.9) years. Among them, 391 (9.6%) died of cardiovascular specific events, and 70 (1.6%) died of diabetes specific events. When MASLD criteria was used, a total of 1508 (40.7%) deaths occurred during follow-up. Among them, 398 (10.4%) died of cardiovascular specific events, and 76 (1.8%) died of diabetes specific events (Table 1). We compared baseline characteristics between alive group and deceased group during follow-up. Compared to alive group, patients in deceased group were more likely to be older, male, Non-Hispanic white/Non-Hispanic black, higher metabolic indexes (including BMI, WC, TG, HbA1c, glucose, insulin, HOMA-IR, and TyG-related indices), higher levels of non-invasive liver fibrosis scores, and more likely to have high sedentary lifestyle, diabetes, and hypertension, while they were less likely to have a higher education (*p* < 0.017). Surprisingly, patients in deceased group were more likely to be a current smoker in patients with MASLD but not with NAFLD. However, there were no significant differences in income, marital status, and HDL-C levels between the groups in patients with either NAFLD or MASLD (Table 2).

**Table 2:** Baseline characteristics of participants with NAFLD or MASLD by mortality status

Population	NAFLD			MASLD		
Mortality status	Alive (*n* = 2206 )	Deceased (*n* = 1466)	*p* value	Alive (*n* = 2048 )	Deceased (*n* = 1508)	*p* value
Age (years)	37.0 (29.0, 46.0)	62.0 (51.0, 68.0)	<0.001	38.0 (30.0, 47.0)	62.0 (50.0, 68.0)	<0.001
Male, n (%)	992 (48.0)	772 (57.6)	<0.001	942 (50.8)	801 (57.7)	<0.001
Race/ethnicity, n (%)			<0.001			<0.001
Non-Hispanic white	680 (67.0)	635 (67.5)		604 (62.5)	645 (67.0)	
Non-Hispanic black	562 (12.3)	402 (12.7)		508 (12.4)	415 (12.9)	
Mexican-American	855 (14.0)	381 (15.4)		829 (15.3)	396 (14.9)	
Other race	109 (6.6)	48 (4.4)		107 (7.2)	52 (5.1)	
Low income, n (%)	800 (20.5)	502 (24.4)	0.223	757 (21.8)	533 (25.8)	0.339
Married, n (%)	1365 (67.6)	900 (69.0)	0.794	1298 (68.9)	915 (68.0)	0.108
College degree, n (%)	276 (23.0)	116 (13.5)	<0.001	245 (21.3)	111 (12.6)	<0.001
Current smoker, n (%)	496 (23.1)	377 (21.9)	<0.001	458 (22.9)	399 (23.0)	<0.001
Sedentary lifestyle, n (%)	701 (24.1)	561 (30.1)	<0.001	681 (25.0)	584 (30.7)	<0.001
BMI (kg/m^2^)	28.2 (24.3, 32.7)	29.5 (26.1, 33.8)	<0.001	28.8 (25.4, 33.1)	29.5 (26.2, 33.8)	<0.001
WC (cm)	95.6 (84.2, 106.2)	103.0 (94.5, 112.3)	<0.001	97.4 (87.9, 107.2)	103.0 (94.7, 112.4)	<0.001
Diabetes, n (%)	242 (8.5)	462 (27.3)	<0.001	245 (9.4)	477 (28.1)	<0.001
Hypertension, n (%)	613 (26.4)	915 (59.6)	<0.001	628 (30.0)	952 (60.3)	<0.001
TG (mg/dL)	124.0 (80.0, 195.0)	156.0 (106.0, 231.0)	<0.001	134.0 (87.0, 206.0)	156.0 (106.0, 231.0)	<0.001
HDL-C (mg/dL)	46.0 (28.0, 54.0)	44.0 (37.0, 54.0)	0.081	44.0 (37.0, 53.0)	44.0 (37.0, 54.0)	0.529
HbA1c (%)	5.3 (5.0, 5.7)	5.7 (5.3, 6.4)	<0.001	5.3 (5.0, 5.7)	5.7 (5.3, 6.4)	<0.001
Glucose (mg/dL)	93.3 (87.4, 100.8)	100.3 (92.8, 118.5)	<0.001	94.2 (88.1, 101.8)	100.6 (92.9, 119.3)	<0.001
Insulin (uU/mL)	11.13 (7.56, 17.79)	14.02 (9.20, 21.68)	<0.001	11.87 (8.18, 18.62)	14.27 (9.36, 21.96)	<0.001
HOMA-IR	2.60 (1.69, 4.41)	3.67 (2.24, 6.56)	<0.001	2.80 (1.86, 4.61)	3.72 (2.29, 6.68)	<0.001
TyG index	8.68 (8.21, 9.19)	9.02 (8.60, 9.53)	<0.001	8.76 (8.31, 9.25)	9.02 (8.61, 9.53)	<0.001
TyG-BMI index	250.53 (202.97, 295.74)	272.68 (231.87,314.93)	<0.001	258.07 (218.37,299.9)	273.02 (232.97,315.06)	<0.001
TyG-WC index	846.12 (705.02.965.43)	949.28 (839.89,1052.09)	<0.001	865.82 (748.96, 978.01)	949.28 (842.54, 1052.48)	<0.001
NFS	−2.63 ( −3.40, −1.72)	−0.99 (−1.98, 0.05)	<0.001	−2.55 ( −3.36, −1.64)	−0.98 ( −1.99, −0.01)	<0.001
NFS >-1.455, n (%)	423 (17.0)	916 (61.7)	<0.001	424 (19.0)	944 (61.9)	<0.001
FIB-4	0.66 (0.48, 0.83)	1.18 (0.84, 1.62)	<0.001	0.67 (0.49, 0.95)	1.19 (0.85, 1.63)	<0.001
FIB-4 > 1.3, n (%)	189 (8.5)	617 (45.3)	<0.001	191 (9.3)	639 (45.4)	<0.001

Data are displayed as the median (interquartile range) or unweighted frequency counts (weighted percentage) as appropriate. The Mann-Whitney test for continuous variables and the Rao-Scott chi-square test for categorical variables were used in this analysis

BMI, body mass index; FIB-4, fibrosis-4 index; HbA1c, glycated hemoglobin; HDL-C, high-density lipoprotein cholesterol; HOMA-IR, homeostasis model assessment of insulin resistance; MASLD, metabolic dysfunction-associated steatotic liver disease; NAFLD, non-alcoholic fatty liver disease; NFS, NAFLD Fibrosis Score; TG, triglycerides; TyG, triglyceride-glucose; WC, waist circumference

### Association between TyG-related indices with all-cause and cause-specific mortality among patients with NAFLD/MASLD

After adjusting for age, sex, and ethnicity using a multivariable Cox regression model (model 1), higher TyG-BMI, and TyG-WC indices were significantly and positively associated with all-cause mortality among individuals with either NAFLD or MASLD. The associations remained significant even after further adjustment for income, marital status, education, smoking, sedentary lifestyle, diabetes, hypertension, plasma HDL-C, and FIB-4 (model 2) (Tables 3 and 4). For TyG-BMI index in NAFLD, adjusted HR (aHR) (95% CI): 1.002 (1.001, 1.003), *p*<0.001; for TyG-WC index in NAFLD, aHR (95% CI): 1.001 (1.001, 1.002), *p*<0.001 (Table 3). For TyG-BMI index in MASLD, aHR (95% CI): 1.002 (1.001, 1.003), *p*<0.001; for TyG-WC index in MASLD, aHR (95% CI): 1.001 (1.000,1.002), *p*<0.001 (Table 4). However, the TyG index was independently associated with an increase in all-cause mortality only in model 1 among individuals with NAFLD/MASLD.

**Table 3:** HRs of TyG-related indices for all-cause and cause-specific mortality among participants with NAFLD.

	Model 1		Model 2	
	HR (95% CI)	*p* value	HR (95% CI)	*p* value
*Outcome: All-cause deaths*				
TyG index	1.201 (1.124, 1.284)	<0.001	1.095 (1.007, 1.190)	0.033
TyG-BMI index	1.003 (1.002, 1.004)	<0.001	1.002 (1.001, 1.003)	<0.001
TyG-WC index	1.001 (1.001, 1.002)	<0.001	1.001 (1.001, 1.002)	<0.001
*Outcome: Cardiovascular specific deaths*				
TyG index	1.270 (1.139, 1.420)	<0.001	1.036 (0.904, 1.187)	0.610
TyG-BMI index	1.004 (1.002, 1.005)	<0.001	1.002 (1.001,1.003)	0.011
TyG-WC index	1.002 (1.001, 1.002)	<0.001	1.001 (1.000, 1.001)	0.017
*Outcome: Diabetes-specific deaths*				
TyG index	1.900 (1.520, 2.380)	<0.001	1.100 (0.817, 1.480)	0.530
TyG-BMI index	1.010 (1.007, 1.012)	<0.001	1.005 (1.002, 1.008)	0.002
TyG-WC index	1.005 (1.003, 1.010)	<0.001	1.002 (1.001, 1.003)	0.005

Survey weight-adjusted multivariable Cox proportional hazard models were performed for all-causes mortality. Competing risk analyses of cause-specific mortality were performed. Model 1 was adjusted for baseline age, sex, and race/ethnicity. Model 2 was further adjusted for income, marital status, education, smoking, sedentary lifestyle, diabetes, hypertension, plasma HDL-C, and FIB-4 in addition to model 1

BMI, Body mass index; CI, confidence intervals; FIB-4, Fibrosis-4 index; HDL-C, high-density lipoprotein cholesterol; HR, hazard ratio; NAFLD, non-alcoholic fatty liver disease; TyG, triglyceride-glucose; WC, waist circumference

**Table 4:** HRs of TyG-related indices for all-cause and cause-specific mortality among participants with MASLD.

	Model 1		Model 2	
	HR (95% CI)	*p* value	HR (95% CI)	*p* value
*Outcome: All-cause deaths*				
TyG index	1.182 (1.106, 1.263)	<0.001	1.068 (0.984, 1.160)	0.117
TyG-BMI index	1.002 (1.001, 1.003)	<0.001	1.002 (1.001, 1.003)	<0.001
TyG-WC index	1.001 (1.001,1.002)	<0.001	1.001 (1.000,1.002)	<0.001
*Outcome: Cardiovascular specific deaths*				
TyG index	1.055 (0.940, 1.220)	0.310	1.059 (0.897, 1.250)	0.500
TyG-BMI index	1.003 (1.001, 1.004)	0.002	1.002 (1.001,1.004)	0.005
TyG-WC index	1.001 (1.000, 1.002)	0.002	1.001 (1.000, 1.002)	0.005
*Outcome: Diabetes-specific deaths*				
TyG index	1.840 (1.476, 2.290)	<0.001	1.077 (0.810, 1.432)	0.610
TyG-BMI index	1.009 (1.006, 1.010)	<0.001	1.004 (1.001, 1.010)	0.013
TyG-WC index	1.004 (1.003, 1.008)	<0.001	1.002 (1.001, 1.003)	0.021

Survey weight-adjusted multivariable Cox proportional hazard models were performed for all-causes mortality. Competing risk analyses of cause-specific mortality were performed. Model 1 was adjusted for baseline age, sex, and race/ethnicity. Model 2 was further adjusted for income, marital status, education, smoking, sedentary lifestyle, diabetes, hypertension, plasma HDL-C, and FIB-4 in addition to model 1

BMI, Body mass index; CI, confidence intervals; FIB-4, Fibrosis-4 index; HDL-C, high-density lipoprotein cholesterol; HR, hazard ratio; MASLD, metabolic dysfunction-associated steatotic liver disease; TyG, triglyceride-glucose; WC, waist circumference

After adjusting for age, sex, and ethnicity using a multivariable Cox regression model (model 1), higher TyG-BMI and TyG-WC indices were also significantly and positively associated with cardiovascular mortality among individuals with either NAFLD or MASLD. The associations remained significant in model 2 (Tables 3 and 4). For TyG-BMI index in NAFLD, aHR (95% CI): 1.002 (1.001,1.003), *p* = 0.011; for TyG-WC index in NAFLD, aHR (95% CI): 1.001 (1.000, 1.001), *p* = 0.017 (Table 3). For TyG-BMI index in MASLD, aHR (95% CI): 1.002 (1.001,1.004), *p* = 0.005; for TyG-WC index in MASLD, aHR (95% CI): 1.001 (1.000, 1.002), *p* = 0.005 (Table 4). However, the TyG index was independently associated with an increase in cardiac-specific mortality only in model 1 among individuals with NAFLD.

Similarly, after adjusting for age, sex, and ethnicity using a multivariable Cox regression model (model 1), higher TyG-BMI and TyG-WC indices were significantly and positively associated with diabetes mortality among individuals with either NAFLD or MASLD. The associations remained significant in model 2 except for TyG-WC index in MASLD (Tables 3 and 4). For TyG-BMI index in NAFLD, aHR (95% CI): 1.005 (1.002, 1.008), *p =* 0.002; for TyG-WC index in NAFLD, aHR (95% CI): 1.002 (1.001, 1.003), *p =* 0.005 (Table 3). For TyG-BMI index in MASLD, aHR (95% CI): 1.004 (1.001, 1.010), *p =* 0.013 (Table 4). For TyG-WC index in MASLD, the *p*-value was close to 0.017, although the statistical significance was not reached. However, the TyG index was independently associated with an increase in diabetes mortality only in model 1 among individuals with NAFLD/MASLD.

### Non-linear trends of TyG-related indices with all-cause mortality among patients with NAFLD/MASLD

**Fig. 1:**
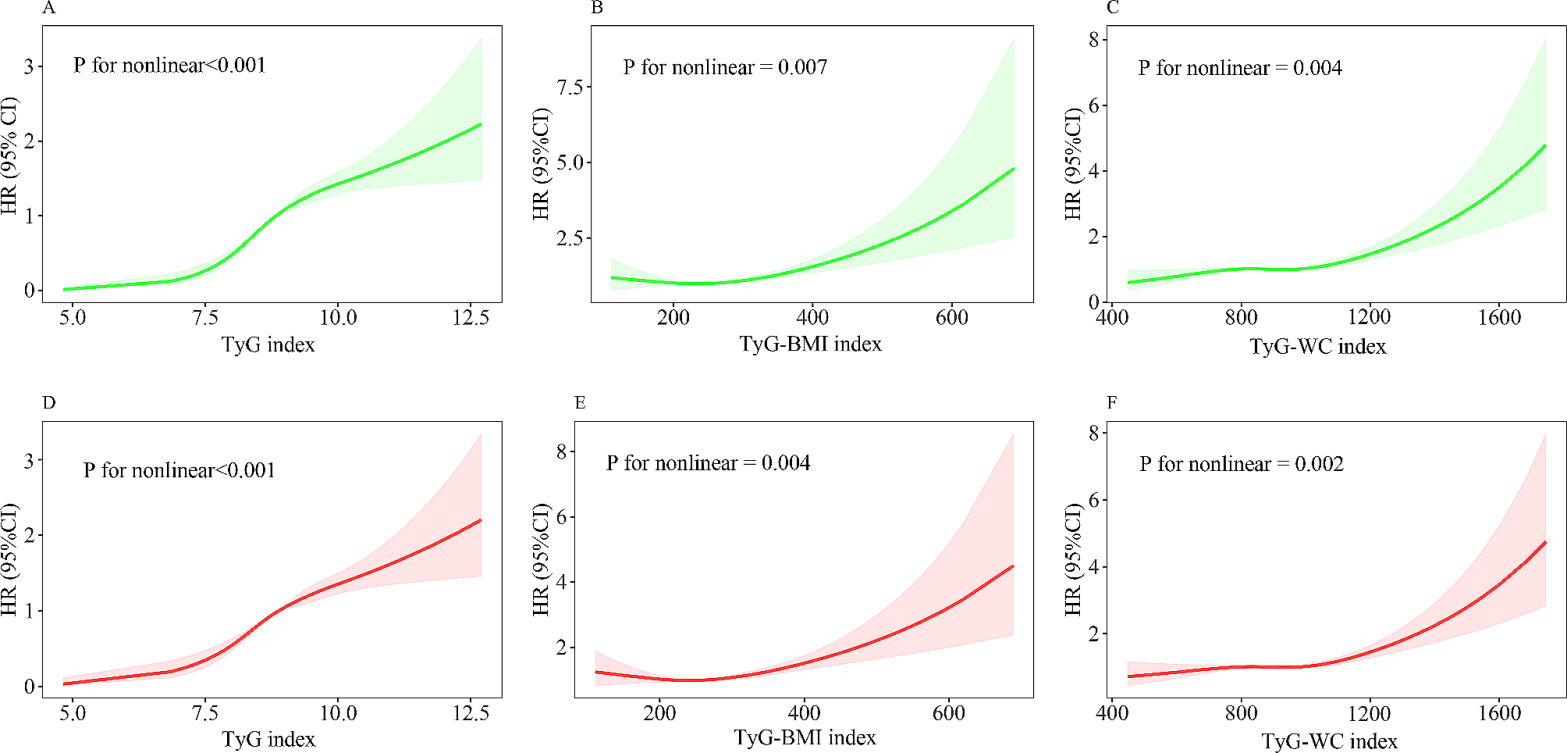
Nonlinear relationship between TyG-related indices and all-cause mortality among NAFLD/MASLD. The relationship was evaluated by RCS after adjustment for age, sex, race, income, marital status, education, smoking, sedentary lifestyle, diabetes, hypertension, plasma HDL-C, and FIB-4 (model 2). The solid lines in the figure represents HRs, and the shaded regions represents the 95% CIs. (A) TyG index in NAFLD; (B) TyG-BMI index in NAFLD; (C) TyG-WC index in NAFLD; (D) TyG index in MASLD; (E) TyG-BMI index in MASLD; (F) TyG-WC index in MASLD. BMI, Body mass index; CI, confidence intervals; FIB-4, Fibrosis-4 index; HDL-C, high-density lipoprotein cholesterol; HR, hazard ratios; MASLD, metabolic dysfunction-associated steatotic liver disease; NAFLD, non-alcoholic fatty liver disease; RCS, restricted cubic spline; TyG, triglyceride-glucose; WC, waist circumference

We conducted RCS to flexibly model and visualize the associations between TyG-related indices with all-cause mortality among patients with NAFLD/MASLD. After adjusting for all covariates in the master analytical model 2 above, a nonlinear trend was observed in the relationships between TyG, TyG-BMI, and TyG-WC indices with the all-cause mortality of the NAFLD population (*p* for nonlinear < 0.001, = 0.007 and = 0.004, respectively) (Fig. 1A–C). Similarly, a nonlinear correlation was observed between TyG, TyG-BMI, and TyG-WC indices and the all-cause mortality of the MASLD population (*p* for nonlinear < 0.001, = 0.004 and = 0.002, respectively) (Fig. 1D–F).

### Subgroup analyses of association between TyG-related indices and all-cause mortality among patients with NAFLD/MASLD

**Fig. 2:**
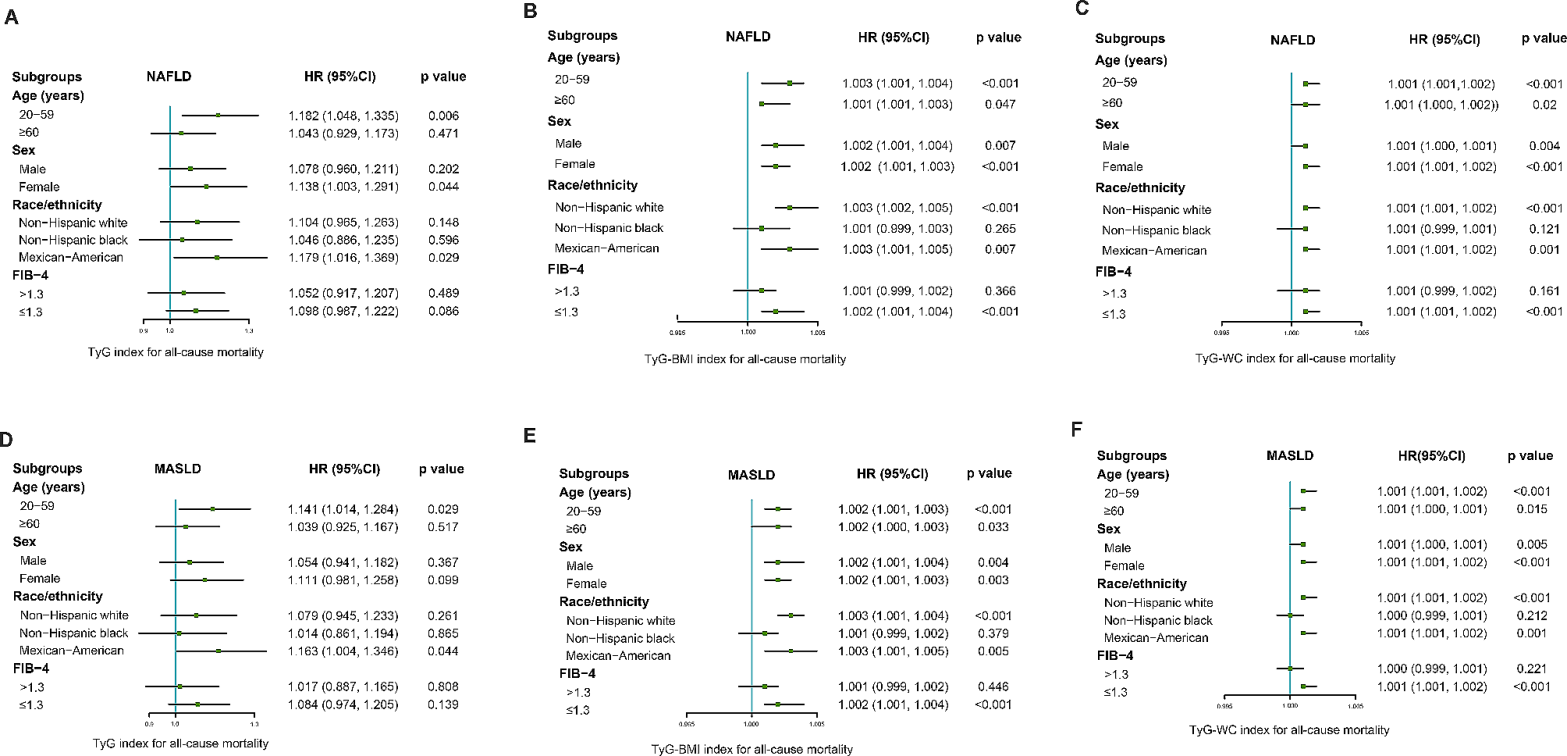
Subgroup analyses of association between TyG-related indices and all-cause mortality among NAFLD/MASLD. Green box means HR value, and the bars on both sides of box mean 95% CI of HR. The adjusted model 2 (age, sex, race, income, marital status, education, smoking, sedentary lifestyle, diabetes, hypertension, plasma HDL-C, and FIB-4) was used in this analysis. (A) TyG index in NAFLD; (B) TyG-BMI index in NAFLD; (C) TyG-WC index in NAFLD; (D) TyG index in MASLD; (E) TyG-BMI index in MASLD; (F) TyG-WC index in MASLD. BMI, body mass index; CI, confidence intervals; FIB-4, Fibrosis-4 index; HDL-C, high-density lipoprotein cholesterol; HR, hazard ratios; MASLD, metabolic dysfunction-associated steatotic liver disease; NAFLD, non-alcoholic fatty liver disease; TyG, triglyceride-glucose; WC, waist circumference .

To further investigate the association between TyG-related indices and all-cause mortality across diverse population, we stratified the NAFLD/MASLD population based on age, sex, race, and the presence of advanced fibrosis (FIB-4 >1.3). After controlling for variables in model 2, stratified analyses revealed that TyG-BMI and TyG-WC indices were found to be significantly associated with increased all-cause mortality in all ages, male and female, non-Hispanic white, Mexican-American, and without advanced fibrosis (Fig. 2B–C, E–F). However, significant correlation between TyG index and all-cause mortality was more likely to occur among individuals who were 20–59 years of age and Mexican-American (Fig. 2A, D). These results suggested that the TyG-BMI and TyG-WC indices were more suitable for predicting all-cause mortality in all ages, male and female, non-Hispanic white, Mexican-American, and without advanced fibrosis among patients with NAFLD/MASLD.

## Discussion

In this prospective cohort study, we preliminary observed the association between a series of TyG-related indices with the all-cause and cause-specific mortality of the NAFLD/MASLD, based on one large-scale, population-based cohort. Higher levels of TyG-BMI and TyG-WC indices are independently associated with an increased risk of all-cause mortality, cardiovascular mortality, and diabetes mortality among participants with either NAFLD or MASLD. The RCS curves displayed non-linear positive associations between TyG-related indices with all-cause mortality of the NAFLD/MASLD population. In addition, the associations between TyG-BMI and TyG-WC indices with all-cause mortality were more pronounced among participants without advanced fibrosis. In brief, our findings indicated that TyG-related indices might be useful surrogate biomarkers for the clinical follow-up management of the NAFLD/MASLD population.

In the general population, NAFLD/MASLD accounts for a substantial proportion, which significantly increases risk for multiple diseases and impairs health outcomes. In line with previous reports, our data showed that the prevalence of NAFLD and MASLD amongst the general population is 35.3% and 34.2%, respectively [1]. Therefore, the identification of potential prognostic and risk factors would improve the management of this population. Patients with NAFLD/MASLD are often accompanied by IR, disordered glucose and lipids metabolism, and activated inflammation [31, 32]. It is widely known that TyG index has been determined to be a simple and reliable tool for detecting IR and evaluating cardiovascular disease risks among the general population [33]. Several noteworthy advances have been made in the application of these surrogate biomarkers to screening NAFLD/MASLD. Wang et al. found that higher baseline TyG indexes or higher excessive TyG exposures were associated with a higher risk of developing NAFLD [34]. Similarly, compared to individuals without NAFLD/MASLD, patients with NAFLD/MASLD were more likely to be higher TyG-related indices in our research. Moreover, deceased patients had higher TyG-related indices than alive patients with either NAFLD or MASLD.

Notably, the TyG index was recently identified to be associated with the prognosis of the population with multiple different diseases, such as coronary heart disease and hypertension, diabetes mellitus, metabolic syndrome, and idiopathic pulmonary arterial hypertension [13, 16, 35, 36]. Meanwhile, the latest evidence suggested that the TyG index with the combinations of adiposity indicators, including but not limited to TyG-BMI and TyG-WC, showed higher predictive performance than the single TyG index in predicting the survivals of patients [35, 37, 38]. Zhan et al. reported that a higher baseline TyG-BMI index was independently associated with an increased risk of cardiovascular disease and all-cause mortality in patients receiving peritoneal dialysis [38]. Wang et al. reported that TyG-WC and TyG-WHtR indices were significantly associated with cardiovascular and diabetes mortality risks among the MetS [35]. However, a limited number of studies have investigated the promising predictive role of the TyG-related indices in NAFLD/MASLD. Whether it would be a surrogate biomarker for health care in the NAFLD/MASLD population, especially in countries with high NAFLD/MASLD burdens, remains unknown.

Therefore, a large-scale, prospective, U.S. population-based cohort was used to investigate the association between TyG-related indices and all-cause mortality as well as cause-specific mortality. Our study suggested TyG-BMI and TyG-WC indices presented strong relationships with all-cause mortality, cardiovascular mortality, and diabetes mortality among patients with either NAFLD or MASLD. The RCS curves displayed non-linear positive associations between the indices with all-cause mortality of the NAFLD/MASLD. In addition, the TyG-BMI and TyG-WC indices seem to have stronger predictive ability in participants without advanced liver fibrosis. Min et al. recently reported the predictive value of TyG-related indices in the mortality outcomes of adults with MASLD using NHANES 1999 to 2018 [39]. However, compared to the study, our study was based on an analysis of the NHANES III (1988–1994), which was the only survey component with liver ultrasonography data and had a longer follow-up period. The presence of hepatic steatosis was determined by abdominal ultrasonography, but not by fatty liver index (FLI), which was initially developed using ultrasound-derived hepatic steatosis data. Since NAFLD/MASLD is closely related to diabetes, we also investigated the correlation between TyG-related indices and diabetes mortality. In addition, our research evaluated the associations between TyG-related indices and long-term all-cause/cause-specific mortalities among participants with both NAFLD and MASLD. These results provided evidence that data generated in the NAFLD can be used interchangeably for MASLD. The TyG-related indices in our study include TyG-BMI that was not present in their study. The subgroup analyses showed that TyG-BMI and TyG-WC indices were more suitable for predicting all-cause mortality in patients without advanced fibrosis. Thus, our study provided more information on the relationship between TyG-related indices and long-term mortality among participants with NAFLD/MASLD.

Despite emerging clinical epidemiological findings have confirmed the performance of TyG-related indices in predicting the onset and progression of diseases, little is known about the underlying biological mechanisms. It is clear that IR is closely associated with endothelial dysfunction, oxidative stress, and inflammatory response of the systemic metabolism [40, 41]. As early indicators of IR, TyG-related indices might highlight the participants’ pro-inflammatory condition and indicate their vulnerability to disease progression and severity [35]. It is noteworthy that one non-negligible question raised by recent studies is what the best cut-off point is for TyG-related indices among different stratified populations [33, 42]. Consequently, further studies are required in order to uncover the adequate cut-off point for novel IR biomarkers associated with long-term mortality in patients with NAFLD/MASLD for various ethnicities and liver fibrosis state.

### Strengths and limitations

This study has the following strengths and limitations. First, this study evaluated the relationships between TyG-related indices with the all-cause as well as cause-specific mortality in patients with both NAFLD and MASLD, and found that TyG-related indices, particularly in TyG-BMI and TyG-WC indices can serve as good prognostic evaluation indices. These results provided evidence that data generated in the NAFLD can be used interchangeably for MASLD. Second, the prospective, population-based study design offers robust evidence linking the TyG-related indices to mortality among NAFLD/MASLD patients. Additionally, we have controlled a series of covariates to determine the independent associations between TyG-related indices with the mortality outcomes of NAFLD/MASLD. Last, the subgroup analysis showed that the TyG-BMI and TyG-WC indices were more suitable for predicting all-cause mortality in patients without advanced fibrosis.

However, several limitations should be noted in this study. First, anthropometric measurements and blood indicators were collected only at baseline. It would be worthwhile to research whether trajectories of TyG-related indices could offer insight into NAFLD/MASLD clinical management. Second, as an observational study, there was some inherent bias such as unmeasured confounders that could not be completely ruled out. Therefore, further studies are needed to confirm our findings. Last, only patients from the U.S. were included in the study, thus the practical clinical application of TyG-related indices in varied races from other countries needs to be further validated.

## Conclusion

In summary, TyG-related indices, particularly in TyG-BMI and TyG-WC indices showed significant correlations with all-cause mortality, cardiovascular mortality, and diabetes mortality among U.S. adults with either NAFLD or MASLD. In addition, the nonlinear positive associations between TyG-BMI and TyG-WC indices with all-cause mortality were more pronounced among participants without advanced fibrosis. The findings in our study demonstrated that TyG-related indices can be used to aid clinicians in making better clinical decisions for NAFLD/MASLD patients during long-term follow-up.

## Data Availability

The datasets generated and/or analysed during the current study are available from the corresponding author upon reasonable request.
